# ^18^F-Fluciclovine PET/CT performance in biochemical recurrence of prostate cancer: a systematic review

**DOI:** 10.1038/s41391-021-00382-9

**Published:** 2021-05-19

**Authors:** Soroush Rais-Bahrami, Jason A. Efstathiou, Catriona M. Turnbull, Stephen B. Camper, Andy Kenwright, David M. Schuster, Andrew F. Scarsbrook

**Affiliations:** 1 Department of Urology, University of Alabama at Birmingham, Birmingham, AL USA; 2 Department of Radiology, University of Alabama at Birmingham, Birmingham, AL USA; 3 O’Neal Comprehensive Cancer Center, University of Alabama at Birmingham, Birmingham, AL USA; 4 Department of Radiation Oncology, Massachusetts General Hospital, Harvard Medical School, Boston, MA USA; 5 Blue Earth Diagnostics Ltd, Oxford, UK; 6 Blue Earth Diagnostics Inc, Burlington, MA USA; 7 Department of Radiology and Imaging Sciences, Emory University, Atlanta, GA USA; 8 Department of Radiology, Leeds Teaching Hospitals NHS Trust, Leeds, UK; 9 Leeds Institute of Health Research, University of Leeds, Leeds, UK

**Keywords:** Prostate cancer, Prostate cancer, Prostate cancer

## Abstract

**Background:**

A systematic literature review of the performance of ^18^Fluorine-fluciclovine PET/CT for imaging of men with recurrent prostate cancer was performed.

**Methods:**

Scientific literature databases (MEDLINE, ScienceDirect and Cochrane Libraries) were searched systematically during Oct 2020 using PRISMA criteria. No limit was put on the date of publication. Prospective studies reporting a patient-level ^18^F-fluciclovine detection rate (DR) from ≥25 patients with recurrent prostate cancer were sought. Proceedings of relevant meetings held from 2018 through Oct 2020 were searched for abstracts meeting criteria.

**Results:**

Searches identified 321 unique articles. In total, nine articles (six papers and three conference abstracts), comprising a total of 850 patients met inclusion criteria. Most studies (*n* = 6) relied on ASTRO-Phoenix Criteria, EAU-ESTRO-SIOG, and/or ASTRO-AUA guidelines to identify patients with biochemical recurrence. Patients’ PSA levels ranged from 0.02–301.7 ng/mL (median level per study, 0.34–4.10 ng/mL [*n* = 8]). Approximately 64% of patients had undergone prostatectomy, but three studies focused solely on post-prostatectomy patients. Adherence to imaging protocol guidelines was heterogeneous, with variance seen in administered activity, uptake and scan times. Overall patient-level DR varied between studies from 26% to 83%, with 78% of studies reporting a DR > 50%. DR was proportional to PSA, but even at PSA < 0.5 ng/mL DR of up to 53% were reported. Prostate/bed DR (*n* = 7) ranged from 18% to 78% and extra-prostatic rates (*n* = 6) from 8% to 72%. Pelvic node and bone lesion DR ranged from 8% to 47% and 0% to 26%, respectively (*n* = 5). ^18^F-Fluciclovine PET/CT was shown to impact patient management and outcomes. Two studies reported 59–63% of patients to have a management change post-scan. A further study showed significant increase in failure-free survival following ^18^F-fluciclovine-guided compared with conventional imaging-guided radiotherapy planning.

**Conclusions:**

^18^F-Fluciclovine PET/CT shows good performance in patients with recurrent prostate cancer leading to measurable clinical benefits. Careful adherence to recommended imaging protocols may help optimize DR.

## Introduction

Prostate cancer is the second most common cancer type worldwide, accounting for almost 1.4 million new cases and approximately 375 000 deaths in 2020 [1]. Despite improvements in primary treatments, as many as 53% of all patients undergoing initial therapy with radical prostatectomy or radiation therapy will experience biochemical recurrence which is characterized by rising levels of prostate-specific antigen (PSA) [2].

In order to facilitate treatment when recurrent lesions are small and most amenable to salvage therapy, early and precise localization of such lesions is critical. [3]. Positron emission tomography (PET) is a well-established molecular imaging modality that is increasingly used to localize recurrent lesions in patients with prostate cancer. Older PET radiopharmaceuticals such as fluorodeoxyglucose and choline have limited performance in some prostate tumor subtypes or in patients with lower PSA recurrence levels [4, 5]. However next-generation radiopharmaceuticals such as ^18^Fluorine (^18^F)-fluciclovine or prostate specific membrane antigen (PSMA)-targeting molecules show encouraging results.

^18^F-Fluciclovine is a synthetic amino acid radiopharmaceutical that is approved by the Food and Drug Administration (FDA) and European Commission for the detection of prostate cancer in patients with elevated PSA following prior treatment. ^18^F-Fluciclovine is in clinical use at over 1300 sites worldwide. The purpose of this work was to systematically review the literature detailing the use of ^18^F-fluciclovine PET/CT in patients with biochemical recurrence of prostate cancer. A key aim was to review the patient populations and imaging protocols as well as the impact both of these factors have had on the detection rates achieved and subsequent clinical outcomes.

## Methods

**Fig. 1:**
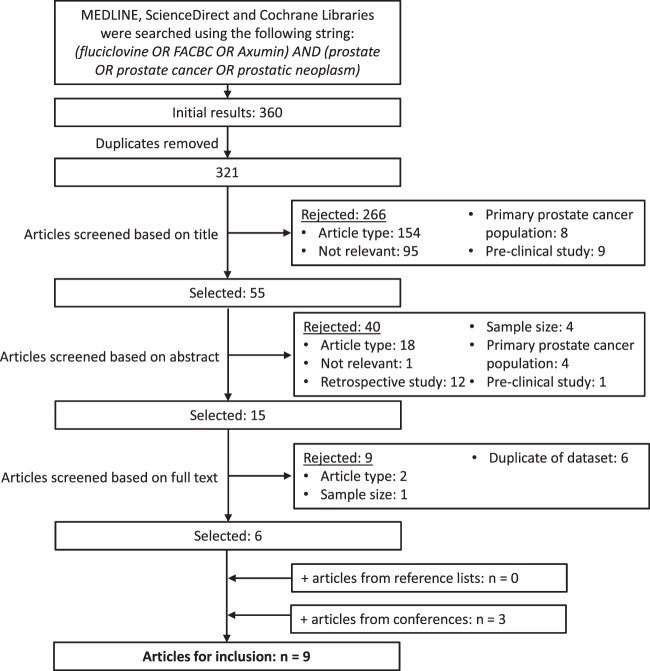
PRISMA flow chart illustrating the systematic literature search process. FABC, Anti-1-amino-3-^18^F-fluorocyclobutane-1-carboxylic acid.

This systematic review was conducted and reported in accordance with the Preferred Reporting Items for Systematic Reviews and Meta-Analyses statement [6]. Scientific literature databases (MEDLINE, ScienceDirect, and Cochrane Libraries) were systematically searched in October 2020 using the search terms detailed in Fig. 1. Searches were limited to journal articles published in English reporting studies in men. No limit was put on the date of publication. All retrospective studies, review articles, practice guidelines, case reports, editorials and letters were excluded. Prospective studies were selected as these were considered the best source of data and as a means of avoiding double counting of data. Studies that reported data from <25 patients with recurrent prostate cancer were excluded to avoid the variability in data from small sample sizes. A minimum data requirement was for a patient-level ^18^F-fluciclovine detection rate in patients with recurrent prostate cancer and all included studies had to either report this directly or have included data from which it could be extrapolated.

Relevant articles were screened, first, on the basis of the title and then on the abstract text as outlined in Fig. 1. The full text of all remaining articles was then reviewed to identify all reports meeting pre-set criteria. Where multiple reports from the same study population were identified, the results of the most recent report were selected for review. The article screening was performed by two independent evaluators (CMT and SBC) and any discrepancies were resolved.

Manual searches of the reference lists of all included articles were conducted using the same screening process as above to seek further studies for inclusion. The proceedings of international urology and nuclear medicine meetings held from January 2018 through October 2020 were also searched for relevant abstracts which may not have been published at the time of the initial literature search. The full presentation corresponding to each abstract was obtained for any that met inclusion criteria. Where the presented data differed from that in the abstract the more recent data (presentation) were used for the purpose of this review.

Biochemical recurrence following radiation therapy is defined by the American Society for Radiation Oncology (ASTRO)/Radiation Therapy Oncology Group Phoenix criteria as a rise of ≥2 ng/mL above the nadir PSA level [7]. A consistent definition is provided by the European Association of Urology (EAU)—European Society for Radiotherapy & Oncology (ESTRO)—International Society of Geriatric Oncology (SIOG) guidelines, which define biochemical recurrence after radiation therapy as an increase in PSA level >2 ng/mL above the nadir [8]. Following radical prostatectomy, biochemical recurrence is defined by the ASTRO/American Urological Association (AUA) guidelines as two consecutive rises in PSA values ≥0.2 ng/mL [9], while the EAU–ESTRO–SIOG guidelines suggest that after radical prostatectomy a PSA level >0.2 ng/mL is associated with residual or recurrent disease [8]. Whether or not these definitions were used to identify patients with recurrent disease was evaluated. Clinical characteristics of the patient populations, image acquisition and reconstruction protocols, detection rates and confirmation method (if any performed and reported) were also evaluated along with any details of post-scan management changes.

Studies were assessed for risk of bias using the Quality Assessment of Diagnostic Accuracy Studies-2 tool which assesses studies based on four domains: patient selection, index test, reference standard, and the study flow and timing [10].

## Results

### Search results

The literature searches identified 321 unique articles. From these, six relevant articles were selected. The main reasons for excluding articles were the article type, the relevance of the study, a primary focus on patients with untreated prostate cancer, or the use of retrospective data. Three further relevant articles were identified from recent conference proceedings. This resulted in nine articles (six papers and three conference presentations) deemed suitable for evaluation in this systematic review [11–19].

As summarized in Table 1, patient selection and the index test methodology were broadly considered to be at low risk of bias. However, when considering the reference standard domain, all nine studies were considered to have some risk. A number of practical and ethical factors limit pathological verification of PET results, particularly in the case of a negative scan, and histopathology is unlikely to accurately determine all sites of metastatic disease. Of the nine studies reviewed, only three used a reference standard (histopathologic correlation, either alone or with follow-up imaging as part of a composite reference standard).

**Table 1:** Risk of bias appraisal of the included studies according to the QUADAS-2 tool.

### Patient populations

Table 2 summarizes the patient populations of the 9 prospective studies included in the review. In total, data from 850 patients with biochemical recurrence who were scanned with ^18^F-fluciclovine were evaluated across the nine studies. Eight of the nine studies (89%) reported the mean age of their cohort and these ranged between 61.8 and 72.0 years. The remaining study reported a median age (68 years). The range of patients’ prescan PSA levels was reported by eight studies and revealed that patients with PSA levels from 0.02 to 301.70 ng/mL were scanned (Table 2). Median PSA data were available for eight of the nine studies and these ranged from 0.34 to 4.10 ng/mL.

**Table 2:** Patient characteristics.

Article	Number of patients receiving ^18^F-fluciclovine	Mean age, years	Pre-scan PSA, ng/mL				Proportion with prior prostatectomy, %
			Mean	SD	Median	Range	
Andriole et al. [15]	213	66.4	4.24	10.22	1.00	0.20–93.5	77
Calais et al. [11]	50	NR^a^	NR	NR	0.48	NR^b^	100
Nanni et al. [12]	89	69.0	6.99	17.50	3.35	0.20–20.72	100
Pernthaler et al. [17]	58	70.1	14.9	33.46^c^	4.10^c^	0.2–230.4	72
Scarsbrook et al. [14]	104	67.5	3.08	4.92	0.79	0.04–28.0	63
Schuster et al. [16]	93	68.0	9.8	31.5	4.00	0.11–301.7	26
Wyndaele et al. [19]	105	72.0	7.05	NR	NR	0.11–47	NR
Lavely et al. [18]	59	69.3	NR	NR	2.30	0.1–91.1	51
Jani et al. [13]	79	61.8	1.67	4.00	0.34	0.02–31.00	100

*NR* not reported.

^a^Median age of 68.0 years reported.

^b^IQR of 0·38–0·83 reported.

^c^Determined from data in Table 2 [17].

Data regarding the patients’ initial disease staging were not widely reported. The proportion of patients with a Gleason score ≥8 ranged from 15–41% across the six studies that reported them [12–15, 17, 18]. Nodal status was reported by only four studies [11–13, 17], which showed that between 19 and 44% of patients had positive nodes.

When considering the patients’ primary therapy, eight of the nine studies reported the proportion of post-prostatectomy patients in their cohort. Of these eight studies, three focused solely on post-radical prostatectomy patients [11–13], while the remaining studies had populations comprising of up to three-quarters of patients with intact prostates (Table 2). Taken together, across all the studies approximately 64% of patients had undergone radical prostatectomy prior to ^18^F-fluciclovine imaging.

Between 8% [14] and 45% [12] of the patients undergoing radical prostatectomy also received radiotherapy around the time of surgery. The number of patients receiving radiotherapy alone was not reported as definitively and the reported proportions varied greatly between studies. Brachytherapy did not appear to be commonly used as an initial therapy, other than in the UK-based FALCON study where 19% of patients received it [14].

Two studies excluded any patient who had received androgen deprivation therapy (ADT) within 3 months of screening [14, 15]. Two further studies reported that all patients receiving ADT had ceased it at the time of the scan [12, 16], and the studies by Jani et al. [13] and Calais et al. [11] had cohorts that were comprised of 38% and 14% patients receiving ADT, respectively. The remaining studies did not report ADT use among their patients.

The majority of the included studies (6/9, 67%) relied on one or more of the ASTRO/RTOG Phoenix, EAU–ESTRO–SIOG, or the ASTRO/AUA definitions to identify patients with biochemical recurrence [11, 12, 14–17], with one also adding a further requirement based on PSA doubling time for post-prostatectomy patients [14]. The remainder of the studies either did not report the PSA criteria or used a different definition [13, 18, 19].

Only one study specified that it was the patient’s first episode of recurrence [14]. However, most reported that they were evaluating patients for suspected biochemical recurrence following primary definitive treatment. Three studies included criteria such as negative conventional imaging to rule out extra-pelvic or bone metastases [13, 15, 16].

### ^18^F-Fluciclovine imaging

Most studies administered ^18^F-fluciclovine at an activity close to the 370 MBq (10 mCi) recommended by the Axumin^®^ (fluciclovine F 18) US Prescribing Information [20], although the reported ranges of administered activity indicate that two studies included patients who received <200 MBq [16, 17]. Two studies did not provide dosing specifics [13, 18]. Seven of the nine (78%) studies conducted the scan with an uptake time in line with standard procedures, i.e., at 3–5 min post-injection [20]. Two studies used a shorter uptake time: Calais et al. [11] had a median uptake time of 2 min (IQR 1–3 min) and patients in the Pernthaler et al study were scanned immediately post-injection [17]. However, the latter was the only study to use a dynamic imaging protocol, and static image acquisition was performed in line with standard procedures. Bed position and scanning times per bed position were not consistently reported, although one study [12] reported a notably shorter time per bed position (2 min/bed) than the other reported values. The most commonly used scanners across the studies were GE Discovery scanners (GE Healthcare), although several different scanner types were used. Details of image reconstruction were not routinely reported by the studies included in this review.

### Detection rates

**Fig. 2:**
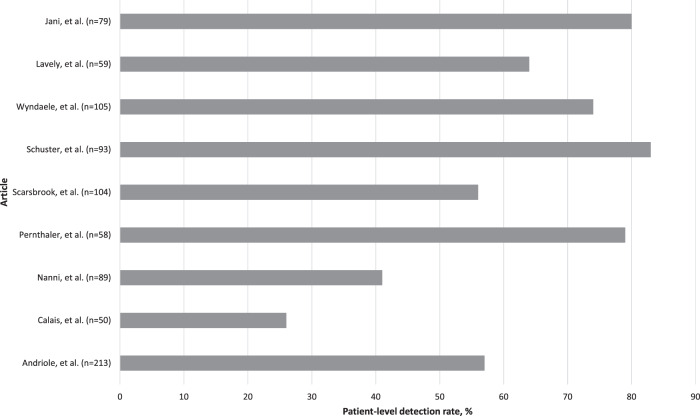
Patient-level detection rates reported by the 9 studies included in our review.

**Fig. 3:**
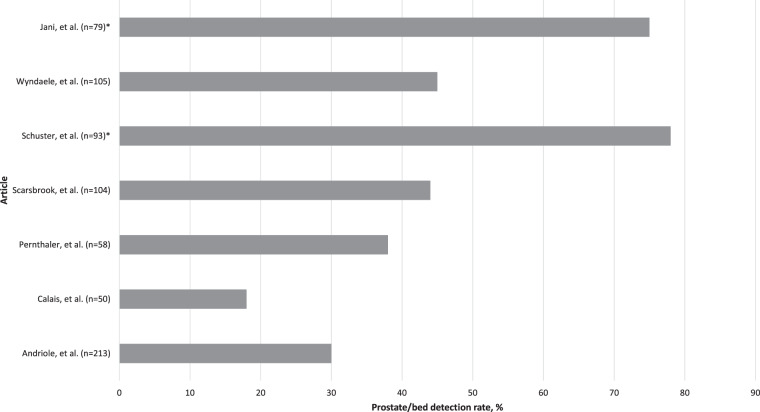
Prostate/bed detection rates reported by 7 studies included in our review. Data either as reported in article or extrapolated* from data provided in text and/or supplementary material.

Overall patient-level detection rates varied across the studies from 26% to 83% (Table 3 and Fig. 2). In total, 78% of studies had a detection rate >50% and 33% of the studies reported a detection rate of more than 75%. As shown in Table 3, seven of the nine (78%) studies provided some breakdown of the detection rate by anatomical region. Prostate/bed detection rates were available for seven studies and ranged from 18% to 78% (Fig. 3), while extra-prostatic detection rates ranged from 8% to 72% (six studies). Pelvic lymph node and bone lesion detection rates were each reported by five studies and ranged from 8% to 47% and 0% to 26%, respectively.

**Table 3:** Reported patient-level and regional detection rates.

Article	*N*	Patient-level detection rate, %	Prostate/bed detection rate, %	Extra-prostatic detection rate, %	Pelvic lymph node detection rate, %	Bone detection rate, %
Andriole et al. [15]	213	57	30	38	NR	11
Calais et al. [11]	50	26	18	8^a^	8	0
Nanni et al. [12]	89	41^b^	NR	NR	NR	NR
Pernthaler et al. [17]	58	79	38	72^c^	47	26
Scarsbrook et al. [14]	104	56	44	25	18	9
Schuster et al. [16]	93	83	78^d^	30^d^	NR	NR
Wyndaele et al. [19]	105	74	45	NR	33	16
Lavely et al. [18]	59	64	NR	NR	NR	NR
Jani et al. [13]	79	80	75^e^	39^e^	34^e^	NR

*NR* not reported.

^a^Determined from reported pelvic LN, extra-pelvic LN, bone and other organ detection rates.

^b^Determined from data reported in Table 5 and number of patients in each category reported in abstract [12].

^c^Determined from data in Table 2 [17].

^d^Determined from data in Table 1 [16].

^e^Determined from data on Slide 12 [13].

### Effect of PSA and prior treatment on detection rates

The lowest overall detection rate reported was from a study that recruited only patients with a PSA level ≤2 ng/mL [11]. Five further studies delineated detection rates by PSA levels (Table 4). Five studies provided data for patients with very low PSA values; they reported that up to 53% of patients had a positive result at PSA levels ≤0.5 ng/mL. Wyndaele et al. [19] and Scarsbrook et al. [14] included data from patients with PSA levels ≤0.2 ng/mL and reported detection rates of 29% (*n* = 7) and 33% (*n* = 18), respectively. In general, detection rates increased with increasing PSA levels, with the data showing that at PSA levels >2 ng/mL, detection rates of 55–97% were achieved. Only one of the studies assessed the impact of PSA doubling time on the outcomes of ^18^F-fluciclovine PET but showed it not to be a significant predictor of scan positivity [11].

**Table 4:** Patient-level detection rates stratified by PSA range.

Article	Overall detection rate, %	Detection rate at PSA ≤ 0.5 ng/mL	Detection rate at PSA < 1 ng/mL	Detection rate at < 2 ng/mL	Detection rate at > 2 ng/mL
Andriole et al. [15]	57	31% (*n* = 81)	36% (*n* = 107)a^a,b^	42% (*n* = 136)^c^	84% (*n* = 77)
Calais et al. [11]	26	27%	27%^b^	26% (*n* = 50)^d^	NR
Nanni et al. [12]	41	NR	21% (*n* = 28)	34% (*n* = 56)^e^	55% (*n* = 33)^f^
Pernthaler et al. [17]	79	20% (*n* = 10)^g^	33% (*n* = 12)^g^	42% (*n* = 19)^g^	97% (*n* = 39)^g^
Scarsbrook et al. [14]	56	29% (*n* = 45)^h^	30% (*n* = 56)^a,h^	30% (*n* = 61)^d,h^	93% (*n* = 43)
Wyndaele et al. [19]	74	53% (*n* = 17)^**i**^	46% (*n* = 24)^a,i^	45% (*n* = 33)^d,**i**^	88% (*n* = 72)^**i**^

*NR* not reported.

^a^Data presented are ≤1.0 ng/mL.

^b^Determined from the reported ≤0.5 and >0.5−1.0 detection rates.

^c^Determined from the reported ≤0.5, >0.5−1.0 and >1.0 − 2.0 detection rates.

^d^Data reported are ≤2.0 ng/mL.

^e^Determined from the reported <1 and 1 – <2 detection rates.

^f^Determined from the 2–<3 and ≥3 detection rates.

^g^Determined from data in Table 2 [17].

^h^Determined from data in Supplementary Table [14].

^i^Determined from data in Table 1 and Fig. 1 [19].

Given the impact of PSA level on the detection rates, it could be expected that patients who were post-radical prostatectomy would have lower detection rates than those with an intact prostate as a consequence of the higher PSA thresholds used to enroll patients who underwent radiotherapy as initial treatment. Only one study [14] presented PSA ranges separately for patients who were post-radical prostatectomy and those with intact prostates, and none of the studies stratified detection data by primary treatment modality. Thus, determining a clear relationship between prostatectomy status and detection rate from these data is not feasible. The study with the highest proportion of patients with an intact prostate achieved the highest overall detection rate [16]. However, similar levels of detection were achieved from populations with a high proportion of patients who had undergone radical prostatectomy [13, 17].

### Use of a reference standard

Only one-third of studies applied a histological and/or clinical reference standard in order to determine the positive predictive value of ^18^F-fluciclovine PET/CT imaging (Table 5). Schuster et al. [16] performed histological sampling and provided region-specific estimates and Nanni et al. [12] evaluated clinical data and imaging history over a 6–24-month period including biopsy data if conducted as part of a patient’s clinical management. A third study (Calais et al. [11]) applied a composite reference standard including histopathology, follow-up imaging, or PSA monitoring to a small proportion of patients (15/50 [30%] patients; five with ^18^F-fluciclovine-positive findings).

**Table 5:** Diagnostic performance of ^18^F-fluciclovine PET/CT compared with clinical and/or histological reference standards.

Article	Region	*n*	Positive predictive value (%)
Schuster et al. [16]	Prostate/bed	91	75
	Extra-prostatic	70	96
Nanni et al. [12]	Overall	89	97
Calais et al. [11]	Overall	5	100

### Post-scan management changes

Three studies reported the effect of the ^18^F-fluciclovine PET/CT on patients’ management plans [13–15]. Two of these documented all types of management changes and showed that following ^18^F-fluciclovine PET/CT, 59–63% of patients experienced a change to their management plan. For a large majority of these patients, the change involved a new treatment modality [14, 15].

The study by Jani et al. [13] compared decisions for prostate cancer radiotherapy that were guided by ^18^F-fluciclovine PET/CT with those made by conventional imaging. Their data show a 35.4% rate of decision changes in patients undergoing ^18^F-fluciclovine PET/CT, which included four patients having planned radiotherapy aborted. Outcome data from the study reported the 3-year failure-free survival (FFS) of those undergoing ^18^F-fluciclovine PET/CT to be 75.5% compared with 63.0% for the patients undergoing conventional imaging (*p* = 0.003). At 4 years the FFS was 75.5% for ^18^F-fluciclovine PET/CT compared with 51.2% for conventional imaging (*p* < 0.001). There was no significant difference in reported toxicity between the two arms, suggesting that treatment to ^18^F-fluciclovine PET-directed volumes was tolerable.

## Discussion

Herein, we conducted a systematic review of the literature to evaluate the growing body of evidence supporting the use of ^18^F-fluciclovine PET/CT for the localization of recurrent lesions in patients with prostate cancer. In this setting we found a small number of studies that prospectively evaluated detection rates. Any inconsistencies in the protocols and methods are discussed and should be set in the context of the very heterogeneous nature of published trials in this area of research. This is evidenced by only 9 out of 321 trials assessed being deemed suitable for inclusion.

As previously reported, ^18^F-fluciclovine detection rates may differ between centers, possibly due to differences in acquisition protocols, scanner type, and/or readers’ experience levels [21, 22]. It may be that unfamiliarity with ^18^F-fluciclovine reporting influenced results, but it has been shown that following limited specific training, naïve readers are able to read ^18^F-fluciclovine images with good agreement [23]. Disparity in detection rates between studies is likely a consequence of differing imaging protocols. For instance, the only two studies [11, 12] reporting a detection rate below 50% both used protocols with shorter than optimal timings. A number of limitations that would have affected image quality have been raised [24] in response to the Calais et al. study [11], including that their median uptake time of 2 min does not follow US FDA-recommended image acquisition guidelines which state that scanning should begin 3–5 min post-injection. Nanni et al. [12] scanned with a time per bed position of 2 min which is shorter than typically used for ^18^F-fluciclovine PET/CT [23].

The impact of patients’ PSA level on ^18^F-fluciclovine detection rates is well established [25] and the data here support increased detection at higher PSA levels. However, the study with the lowest mean and median PSA actually reported one of the highest detection rates (80%) [13]. Moreover, two studies show that at PSA ≤ 0.2 ng/mL, approximately one-third of patients had a positive result providing support to the increasing evidence for good performance of ^18^F-fluciclovine at low or even undetectable PSA levels, and the suggestion that it may benefit patients to conduct ^18^F-fluciclovine PET/CT at lower PSA thresholds than commonly used in current practice [14, 19, 26–28].

The impact a patient’s primary therapy has on the PSA threshold for defining recurrence and, subsequently, recruitment into trials such as those reviewed here is a likely contributor to the variance in detection rates observed across trials with PSA-dependent PET radiopharmaceuticals which do not control for patients’ treatment history. A recent secondary analysis [27] of the LOCATE study [15] shows ^18^F-fluciclovine patient-level detection rates are higher in men with intact prostates (84%) than in those who had radical prostatectomy (49%) with the rates in the prostate/bed thought to account for the difference owing to the similar extra-prostatic detection rates in patients with and without prostates (37% vs 38%, *p* = 1.00). In this review, the varying rates of detection in the prostate/bed (18–78% across 7 studies) might be a consequence of the proportion of post-prostatectomy patients, although this cannot be concluded from the available data which are limited by lack of reports from cohorts solely with intact prostates.

A number of the studies reviewed here compared the performance of ^18^F-fluciclovine with another radiopharmaceutical [11, 12, 16, 17]. Nanni et al. [9] and Schuster et al. [16] compared with established imaging agents, ^11^C-choline or ^111^In-capromab pendetide, respectively and both observed superior performance with ^18^F-fluciclovine. Calais et al. [11] and Pernthaler et al. [17] compared ^18^F-fluciclovine with ^68^Ga-PSMA-11 and showed that overall detection rates were higher with ^68^Ga-PSMA-11 (56% and 83%, respectively) than with ^18^F-fluciclovine (26% and 79%, respectively). ^68^Ga-PSMA-11 provided improved detection in lymph nodes and distant metastases, but ^18^F-fluciclovine offered superior detection of local recurrence in the prostate/prostate bed. Pernthaler et al. noted that ^18^F-fluciclovine is almost equivalent to ^68^Ga-PSMA-11 in the detection of distant metastases and suggests a potential advantage of ^18^F-fluciclovine in the detection of curable localized disease in close proximity to the urinary bladder, where ^68^Ga-PSMA-11 has limited utility due to accumulation in the bladder.

As shown here, there is mounting evidence for a role for ^18^F-fluciclovine in guiding therapy decisions across all treatment modalities [14, 15], and the impact on radiotherapy planning is well researched. Similar to ^18^F-fluciclovine-guided rates of change to radiotherapy plans reported by recent secondary analyses of LOCATE and FALCON (48% and 40%, respectively) [29, 30], Jani et al. [13] showed that 28/79 (35%) patients had a radiotherapy decision change following ^18^F-fluciclovine PET/CT. Importantly, Jani et al. include follow up data to provide the first ever report of a significant increase in FFS resulting from ^18^F-fluciclovine-guided salvage radiotherapy with no significant difference in toxicity.

There are a few limitations to this analysis. As discussed above, the literature provides a very heterogeneous mix of studies and our analysis was limited by inconsistent reporting of data and imaging protocols between studies. Although nine studies met our inclusion criteria, this resulted in an overall sample size of only 850 patients. Very few of these 850 patients had histological verification of their imaging findings preventing any meaningful conclusions being drawn on formal diagnostic metrics beyond the detection rates reported here. Although higher specificity is noted in extraprostatic lesions compared with the treated prostate, perhaps because of overlap between malignancy, benign hyperplasia and prostatitis, prior histologically confirmed data demonstrate good diagnostic performance of ^18^F-fluciclovine-PET/CT in patients with recurrent prostate cancer [25]. The impact of PSA doubling time on ^18^F-fluciclovine detection rates was not well explored, although data in the literature suggest there to be no significant effect [31–33]. Likewise, few studies stratified detection by prior or ongoing therapy, which would have been informative given the influence PSA has on detection rates. There was a paucity of data on the patients’ use of ADT. Current ADT use needs to be considered for any PET radiopharmaceutical given the well-established inhibitory effect on the uptake of choline-based radiopharmaceutical in patients with androgen-sensitive prostate cancer and the potential downregulation of PSMA expression with prolonged ADT use that may impact PSMA-targeting radiopharmaceuticals [34–37].

In summary, this systematic review provides support to the growing role for ^18^F-fluciclovine PET/CT in the localization of recurrent lesions in patients with prostate cancer, with patient-level detection rates of up to 83% observed. ^18^F-Fluciclovine PET/CT provides good rates of detection across wide PSA ranges and can support management decisions that bring about measurable clinical benefit to patients.

## Data Availability

The datasets generated during the current study are available from the corresponding author on reasonable request.
